# Sodium Content in Packaged Foods by Census Division in the United States, 2009

**DOI:** 10.5888/pcd12.140500

**Published:** 2015-04-02

**Authors:** Alexandra K. Lee, Linda J. Schieb, Keming Yuan, Joyce Maalouf, Cathleen Gillespie, Mary E. Cogswell

**Affiliations:** Author Affiliations: Alexandra K. Lee, Emory University, Atlanta, Georgia; Keming Yuan, Joyce Maalouf, Cathleen Gillespie, Mary E. Cogswell, Centers for Disease Control and Prevention, Atlanta, Georgia.

## Abstract

Excess sodium intake correlates positively with high blood pressure. Blood pressure varies by region, but whether sodium content of foods sold varies across regions is unknown. We combined nutrition and sales data from 2009 to assess the regional variation of sodium in packaged food products sold in 3 of the 9 US census divisions. Although sodium density and concentration differed little by region, fewer than half of selected food products met Food and Drug Administration sodium-per-serving conditions for labeling as “healthy.” Regional differences in hypertension were not reflected in differences in the sodium content of packaged foods from grocery stores.

## Introduction

Excess sodium intake is a major preventable risk factor for hypertension (1,2). More than 90% of US adults consume more sodium than recommended (2,3). Recent studies highlighted the challenges of eating low-sodium diets, given the current availability of commercially packaged food products (4,5). Hypertension prevalence varies by geographic region (6). However, it is unclear if regional variation in sodium consumption exists, and, if so, whether that variation is due to regional differences in sodium content of packaged foods (7,8). The purpose of this study was to investigate regional differences in the sodium content of packaged food products sold in US grocery stores.

## Methods

The 2009 product-level point-of-sales Nielsen ScanTrack database (www.nielsen.com/us/en.html) captures all branded products sold in US grocery stores with annual sales of $2 million or more (not including warehouse-type stores and Walmart) and includes 52 markets in 9 US census divisions. For these analyses, 3 census divisions — South Atlantic, East North Central, and Pacific — representing approximately 50% of the US population were chosen to reflect places with high (South Atlantic), medium (East North Central), and low (Pacific) prevalence of hypertension (6).

We identified products in the 10 food categories that contribute the most sodium to the US diet (9). We obtained nutritional information for those products in the top 80% or with greater than 1% of sales in each census division. A detailed description of nutrient data collection methods are published elsewhere (5). To allow comparison of products of different sizes, we estimated the equivalized unit sales, weighted in ounces, calculated as unit sales × unit size in ounces. We then calculated the mean and standard deviation of sodium content in each food category in milligrams (mg) per serving, mg per kilocalorie (density), and mg per 100 grams (concentration). Sodium density accounts for variation in the energy value of each product. Because sodium and kilocalorie consumption are generally highly positively correlated, use of sodium density has been proposed as a way to compare the sodium content of foods with the same amount of calories (4). Weighted *t* tests were used to determine differences between census divisions. The equivalized, sales-weighted proportion of products in each food category meeting Food and Drug Administration (FDA) sodium limits for foods using the “healthy” label claim (ie, <600 mg of sodium/serving for meals and <480 mg/serving for individual foods) was calculated (10). All analyses used grocery product sales and nutrition facts panel data; actual sodium consumption was not measured. We used SAS-callable SUDAAN version 9.3 (RTI, International).

## Results

Out of 3,974 products identified, 3,876 products from the Nielsen sales database were matched with nutrition facts panel information. The sales-weighted mean sodium density varied from 1.34 (standard deviation [SD], 0.65) mg/kcal for savory snacks in the Pacific division to 18.89 (SD, 24.0) mg/kcal for soup in the South Atlantic division (Table 1). Although there were several significant pairwise differences between regions, no clear pattern emerged. Mean sodium density was highest in the East North Central for 4 of the 10 food categories (poultry, cheese, pasta mixed dishes, and meat mixed dishes). In the South Atlantic, mean sodium density was highest for 3 food categories (bread, soup, and savory snacks), and in the Pacific mean sodium density was highest for the remaining 3 food categories (cold cuts, pizza, and sandwiches). Results for sodium concentration were similar to those for sodium density.

**Table 1:** Sales-Weighted Distribution of Sodium Content in Packaged Foods Sold, by the Top Food Categories Contributing to Sodium Consumption, by Census Division, United States, 2009

Food category	No. of Products			Mean (SD) Sodium Density, mg/kcal			Mean (SD) Sodium Concentration, mg/100 g		
	East North Central	South Atlantic	Pacific	East North Central	South Atlantic	Pacific	East North Central	South Atlantic	Pacific
Bread	339	220	243	1.92^a^ (0.35)	1.93 (0.43)	1.84 (0.33)	488.3 (81.9)	477.9 (94.2)	473.8 (76.8)
Cold cuts	267	261	196	5.71^b^ ^,c^ c (3.46)	6.59 (3.81)	7.01 (3.77)	970.6 (359.3)	975.8 (377.7)	1,011.4 (389.1)
Pizza	164	100	90	2.21^d^ (0.31)	2.28 (0.36)	2.35 (0.38)	544.8^d^ (81.6)	565.5 (94.5)	581.7 (90.8)
Poultry	28	22	11	2.82 (2.49)	1.92 (1.77)	1.93 (0.67)	274.9 (177.9)	196.3 (140.3)	274.6 (104.2)
Soup	175	180	171	17.29 (24.1)	18.89 (24.0)	17.59 (22.7)	407.2 (203.0)	392.8 (208.1)	469.5 (393.8)
Sandwiches	122	109	73	2.11 (0.50)	2.18 (0.54)	2.32 (0.49)	524.4^d^ (137.0)	564.5 (152.1)	587.8 (121.3)
Cheese	321	284	201	3.25^e^ (1.60)	3.16^f^ (1.55)	2.54 (1.35)	993.9^g^ (394.9)	981.4^h^ (391.8)	810.3 (335.4)
Pasta mixed dishes	166	165	148	3.17^i^ (1.07)	2.99 (0.87)	2.86 (0.92)	480.9 (416.0)	437.2 (316.7)	428.0 (357.6)
Meat mixed dishes	91	98	70	3.38 (1.14)	3.08 (1.06)	3.25 (0.89)	423.8 (138.4)	423.8 (151.5)	422.8 (120.5)
Savory snacks	389	333	208	1.43 (0.84)	1.45 (0.85)	1.34 (0.65)	701.5 (313.4)	706.0 (312.6)	687.2 (278.8)

Abbreviation: SD, standard deviation.

a *P* = .03 for comparison of East North Central and Pacific.

b *P* = .04 for comparison of East North Central and Pacific.

c *P* = .03 for comparison of East North Central and South Atlantic.

d *P* = .01 for comparison of East North Central and Pacific.

e *P* = .003 for comparison of East North Central and Pacific.

f *P* = .007 for comparison of South Atlantic and Pacific.

g *P* = .004 for comparison of East North Central and Pacific.

h *P* = .009 for comparison of South Atlantic and Pacific.

i *P* = .02 for comparison of East North Central and Pacific.

More than 70% of pizzas, pasta mixed dishes, and meat mixed dishes and 50% to 70% of cold cuts, soups, and sandwiches exceeded FDA “healthy” labeling standards for sodium, whereas less than 10% of breads, savory snacks, and cheeses did (Figure). Few significant differences were seen between markets and are not presented here.

**Figure:**
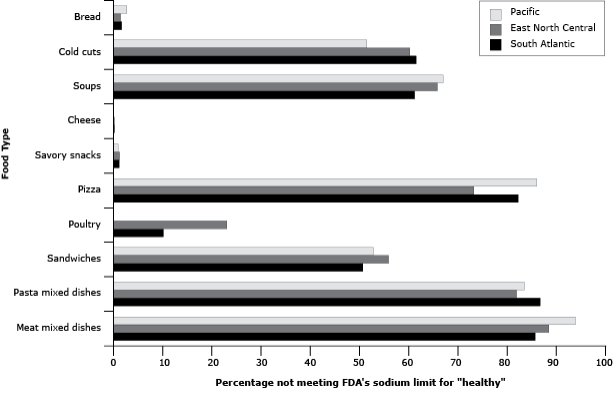
Percentage of packaged food products sold with sodium content higher than the Food and Drug Administration limit for “healthy” food (ie, ≤480 mg per serving for breads, cold cuts, soups, cheese, and savory snacks) or meal (ie, ≤600 mg per serving for pizza, poultry, sandwiches, pasta mixed dishes, and meat mixed dishes), by US Census Division, 2009. Percentages are based on equivalized, sales-weighted estimates (weighted by ounce) to allow comparison of products by common units. **Table:** 

Food type	% Not Meeting FDA Sodium Limit for “Healthy”		
	East North Central	Pacific	South Atlantic
Breads	1.4	2.7	1.7
Cold cuts	60.2	51.6	61.5
Soups	65.8	67.0	61.2
Cheese	0.1	0	0.2
Savory snacks	1.3	0.9	1.1
Pizza	73.2	86.0	82.3
Poultry	23.0	0	10.1
Sandwiches	55.9	52.8	50.7
Pasta mixed dishes	82.0	83.5	86.7
Meat mixed dishes	88.5	93.9	85.6

## Discussion

Few differences in sodium density or concentration of packaged food products, as measured by grocery sales, were found across census divisions. In all 3 divisions, 50% or more of products sold in most food categories exceeded the sodium-per-serving conditions for a “healthy” food.

Although most of the regional differences found did not have a clear direction or contributor, some may relate to regional variations in the popularity of specific types of products within a food category. For example, in a post-hoc analysis, compared with the Pacific division, the South Atlantic and East North Central divisions had higher unit sales of “American cheese” (both cheese and cheese product), which has nearly double the sodium density and sodium concentration of other cheeses. However, because many of the top-selling packaged food products in each region were national brands, regional variation in sodium content of available products may be limited.

Although this study is unique in examining sodium content at the US census division level, we acknowledge several limitations. First, the Nielsen ScanTrack database does not capture all grocery sales in the United States, although it likely provides a representative sample. Second, we did not adjust for multiple comparisons; therefore, some observed differences may be due to chance. Finally, the data indicate sales of products, not consumption. However, in one study, nutrient intake estimated on the basis of sales data was comparable to average self-reported dietary intake from the New Zealand National Nutrition Survey (11).

Despite limitations, these data support recent findings that suggest that meeting sodium recommendations may be difficult in the current food environment, regardless of location (4,5). In all 3 census divisions, the similarly narrow distributions of sodium density in most food categories are indicative of the lack of variation in sodium content. If differences in the food environment are contributing to regional variation in hypertension, it is likely not through variation in the sodium content of packaged food products sold in the grocery store.
