# Erratum, Vol. 12, April 2 Release

**DOI:** 10.5888/pcd12.140500e

**Published:** 2015-09-03

In the article “Sodium Content in Packaged Foods by Census Division in the United States, 2009,” a recommendation by the Food and Drug Administration was incorrectly stated. A sentence in the article previously read, “The equivalized, sales-weighted proportion of products in each food category meeting Food and Drug Administration (FDA) sodium limits for foods using the ‘healthy’ label claim (ie, <600 mg of sodium/serving for meals and <480 mg/serving for individual foods) was calculated.” However, the correct recommendation to receive the “healthy” label claim was ≤600 mg of sodium per serving for meals and ≤480 mg of sodium per serving for individual foods.

The corrections were made to our website on August 21, 2015, and appear online at http://www.cdc.gov/pcd/issues/2015/14_0500.htm. We regret any confusion or inconvenience these errors may have caused.

